# Improved Temporal Trends of Vaccination Coverage Rates in Childhood after the Mandatory Vaccination Act, Italy 2014–2019

**DOI:** 10.3390/jcm10122540

**Published:** 2021-06-08

**Authors:** Michela Sabbatucci, Anna Odone, Carlo Signorelli, Andrea Siddu, Francesco Maraglino, Giovanni Rezza

**Affiliations:** 1 Department Infectious Diseases, National Institute of Health, 00161 Rome, Italy; 2 Ministry of Health, Directorate General Health Prevention, Communicable Diseases and International Prophylaxis, 20379 Rome, Italy; a.siddu@sanita.it (A.S.); f.maraglino@sanita.it (F.M.); g.rezza@sanita.it (G.R.); 3 Department of Public Health, Experimental and Forensic Medicine, University of Pavia, 27100 Pavia, Italy; anna.odone@unipv.it; 4 School of Medicine, Vita-Salute San Raffaele University, 20132 Milan, Italy; signorelli.carlo@hsr.it

**Keywords:** mandatory programs, vaccination coverage, primary prevention, Italy, childhood vaccination

## Abstract

Maintaining high vaccine coverage (VC) for pediatric vaccinations is crucial to ensure herd immunity, reducing the risk of vaccine-preventable diseases (VPD). The Italian vaccination Law (n. 119/2017) reinforced mandates for polio, diphtheria, tetanus, and hepatitis B, extending the mandate to pertussis, *Haemophilus influenzae* type b, chickenpox, measles, mumps, and rubella, for children up to 16 years of age. We analyzed the national temporal trends of childhood immunization rates from 2014 to 2019 to evaluate the impact of the mandatory reinforcement law set in 2017 as a sustainable public health strategy in Italy. In a 3-year period, 9 of the 10 compulsory vaccinations reached the threshold of 95% and VC for chicken pox increased up to 90.5%, significantly. During the same period, the recommended vaccinations (against meningococcus B and C, pneumococcus, and rotavirus) also recorded a significant increase in VC trends. In conclusion, although the reinforcement of compulsory vaccination generated a wide public debate that was amplified by traditional and social media, the 3-year evaluation highlights positive results.

## 1. Introduction

In Italy, selected childhood vaccinations have been mandatory by law nationally since 1888 (Law of the Kingdom of Italy 22 December 1888 for smallpox, Law n. 891/1939 for diphtheria, Law n. 292/1963 for tetanus, Law n. 51/1966 for polio and Law n. 165/1991 for hepatitis B). However, vaccine coverage (VC) decreased in recent years [1,2], both at the national and regional level, due to several factors, e.g., heterogeneity in the vaccination policies, limited effectiveness of the sanctioning system, low risk perception of infectious diseases, confidence in the availability of antimicrobial therapies, scientific misinformation, increased vaccine hesitancy [3,4,5]. As a possible consequence, large measles outbreaks occurred in 2017–2018 in Italy, threatening public health.

Therefore, the Italian government, supported by the opinion of technical bodies and the scientific community, introduced the Decree Law n. 73/2017 (modified by the Law n. 119/2017), which extended mandatory vaccinations from 4 to 10 for children up to sixteen years of age and for unaccompanied foreign minors. The mandates included vaccines against diphtheria, hepatitis B, polio, and tetanus, which were already compulsory, adding vaccines against Haemophilus influenzae type b and pertussis (included in the hexavalent vaccine), chickenpox, measles, mumps, and rubella (administered through combined monovalent plus trivalent vaccine or through tetravalent vaccines).

These vaccinations are actively offered throughout the country and are free of charge for infants between 3 and 24 months of age and for children around 5–6 years of age. Each vaccination is compulsory for those born from 2001 onwards, with the exception of the vaccination against chickenpox, which is compulsory for those born since 2017. The compulsory model for the trivalent vaccination against measles, mumps, and rubella is subject to revision every three years, based on epidemiological data and on VC levels achieved.

Prior to the law on mandatory vaccination, the National Vaccine Prevention Plan 2017–2019 [6] issued by the Ministry of Health recommended also vaccination against human papillomavirus (HPV) at 11 years of age, *Neisseria meningitidis* serogroups ACW135Y and *Streptococcus pneumoniae* for those born from 2012 onwards, and *N. meningitidis* serogroup B and rotavirus for those born since 2017.

Access to kindergarten and school is denied for those defaulting from the national mandatory immunization program and penalties have been provided for uncompliant parents or tutors. This reinforced obligation aimed at reducing the health and social costs [7,8] related to vaccine-preventable diseases (VPD) and possible associated sequelae in the young population. Besides, the law guarantees equal access to the Italian vaccination services for infants and children, avoiding discriminations by region or country of origin. However, the social impact of mandatory vaccination policies remains controversial [9,10]. As the relationship between mandatory immunization policy and VC levels is still unproven in Italy [11,12], we aimed to analyze the national VC trends for the mandatory vaccinations, unravelling possible contributing factors.

## 2. Materials and Methods

We collected anonymized and aggregated data on the number of vaccinations, stratified by age range and vaccine antigen, administered by the Local Health Authorities (LHA) through the all Italian Regions and Autonomous Provinces (R/AP) in Italy. Then, we calculated the VC rates by dividing the absolute number of the vaccinations administered to children per each age group (24 months or 6 years of age) and R/AP by the number of the resident population with the same age (same birth cohort) according to data obtained from the Italian R/AP. We used polio and measles as usual proxy for the hexavalent and trivalent vaccinations, respectively, since in Italy these vaccines are administered in 6-in-1 mostly and 3-in-1 only vaccine formulations. 

We analyzed the data at 24 months and at 6 years of age. Chi-square test was performed on proportions for the years 2019 vs. 2016 by using SPSS statistical program, version 12.0 for Windows (IBM, Armonk, NY, USA). Analysis findings were considered to be statistically significant at a 2-tailed *p* value of ≤0.05.

## 3. Results

### 3.1. Mandatory Vaccinations

In the period 2014–2016, polio VC rates declined (Table 1) at both the time points (year 2016 vs. 2014, polio at 24 months: −1.38%, at 6 years: −3.44%), while measles VC rates fluctuated at the 24 month-time point and markedly decreased at the 6-year time point (−5.48%). At the 24 month-time point, VC rates for chickenpox increased by 9.42%.

Compared to 2016, all VC rates for mandatory vaccinations and chickenpox, both at 24 months and 6 years of age increased in 2019, significantly (*p* ≤ 0.001; Table 1). Polio VC rates at 24 months rose by 1.68%, and at 6 years by 2.88%. Measles VC rates increased at 24 months by 7.23%, at 6 years by 5.34%. At the 24 month-time point, VC rates for chickenpox increased by 44.44% (Table 1).

In particular, in 2017 compared to 2016, VC rates improved for polio (at 24 months: +1.27%, at 6 years: +2.95%) and measles (at 24 months: +4.58%, at 6 years: +3.5%) vaccinations. On the contrary, at the 24 month-time point, VC rates for chickenpox slightly decreased by 0.44%.

In 2018 compared to 2017, VC rates further improved for the polio (at 24 months: +0.49%, at 6 years: +2.02%) and measles (at 24 months: +1.38%, at 6 years: +3.46%) vaccinations. At the time point of 24 months, chickenpox VC rates increased by 28.61%.

In 2019 compared to 2018, interestingly, VC rates for polio vaccination remained stable at 24 months and decreased (−2.09%) at 6 years of age. A similar trend was observed for measles vaccination uptake, which improved slightly (+1.27%) at 24 months and then decreased (−1.62%) at 6 years of age, reaching the level registered in 2014. At 24 month-time point, chickenpox VC rates increased further by 16.27%.

### 3.2. Recommended Vaccinations

In 2014–2016, VC rates for the recommended vaccinations increased (*N. meningitidis* serogroup C rose by 6.73%, *S. pneumoniae* by 0.89%). Data for the other vaccinations were not available, neither for 2014 nor 2015 (Table 2).

At 24 months, VC rates increased more in 2019 than in 2016, with the exception of *N. meningitidis* serogroup C (−1.23%). Significantly, VC rates for *N. meningitidis* serogroup B rose by 54.26%, *N. meningitidis* serogroup ACW135Y by 28.3%, *S. pneumoniae* by 3.65% and rotavirus by 15.6% (*p* ≤ 0.001; Table 2).

Particularly, as for the mandatory vaccinations, at the 24 month-time point, VC rates improved markedly after the Law n. 119/2017 for vaccinations against *N. meningitidis* serogroup B, C and ACW135Y, (year 2017 vs. 2016: +23.85%, +1.97%, +10.28%, respectively) (Table 2) and further remarkable increases were registered during the year 2018 (year 2018 vs. 2017: +7.49%, +2.29%, +17.26%, respectively). 

In 2019, VC rates against *N. meningitidis* serogroup B increased significantly by 22.92% compared to the previous year, while VC rates against *N. meningitidis* serogroup C and ACW135Y decreased significantly (−5.49%) or remained almost stable, respectively.

VC rates for vaccination against *S. pneumoniae* increased by 2.55% in 2017 compared to 2016, and then slightly increased in both 2018 and 2019. 

VC rates for vaccination against rotavirus increased by 3.81% in 2017 vs. 2016, and then by 5.08% in 2018 and 6.71% in 2019 compared to the previous year.

Overall, the VC rate threshold (95%) for polio in children at 24 months of age was reached by 9 R/AP in 2016, and this number increased to 12 R/AP in 2017, and to 14 R/AP, both in 2018 and 2019.

In 2016, there was no R/AP with a VC rate for measles ≥95% in children at 24 months of age, while there was 1 region both in 2017 and in 2018, and this number increased to 9 R/AP in 2019.

Among the recommended vaccinations, anti-pneumococcal vaccination reached ≥95% coverage rates in children at 24 months of age in 1 region only in 2016, while this number increased to 3 R/AP in 2017 and it was maintained in 3 R/AP in the following years. 

In 2016–2019, in children at 5–6 years of age, VC rates for polio reached the ≥95% threshold in 1 region in 2017 and in 3 R/AP in 2018, while for measles, VC rates reached the threshold in 1 region only in 2018. A decrease in the number of R/AP with a VC rate ≥95% was shown in 2019 for both these vaccinations.

## 4. Discussion

Vaccination is the most cost-effective prevention tool in the fight against infectious diseases and one of the greatest successes of public health in modern history. VPD morbidity and mortality rates decreased dramatically worldwide due to mass immunization programs. In Italy, assuming vaccination as the only contributing factor, over 4 million cases of 10 VPD were estimated having been avoided in the last century [13]. Therefore, evidence-based data continue to support the value of universal vaccination programs.

The Italian R/AP provide citizens with universal coverage for health care, and mandatory vaccinations are offered free of charge to anyone included in the vaccination schedule throughout the country, assuring the equity and quality of health services.

A strengthened political commitment toward the achievement of adequate VC rates started in 2014 and brought forth the release of an innovative and updated National Immunization Prevention Plan (PNPV) 2017–2019, to encourage an increase in vaccine confidence and uptake. The Ministry of Health offered new vaccines, defined further target populations, implemented electronic immunization registries and dedicated training for healthcare professionals, introduced economic sanctions for physicians not recommending vaccinations and supported new laws to limit pre-school admissions to vaccinated children. However, VC rates decreased progressively until 2016 [11,12]. Multiple factors contributed to this decline. Vaccine hesitancy, which was one of these factors, comes from a mistaken set of beliefs about risk/benefit, which still leads to misconceptions in Italy [14]. 

An early assessment of the impact of the Law n. 119/2017 on vaccine uptake was encouraging [15,16]. VC rates increased significantly in 2019 compared to the year 2016 before the introduction of the law. Similarly, VC for all the recommended vaccinations increased significantly (with the exception of vaccination against *N. meningitidis* serogroup C that slightly declined in 2019) after the reinforcement of the mandatory law. We suggest that vaccine mandates for children may have generated public attention and discussion that increased to some extent the adherence also to recommended vaccination, that are offered actively at the vaccination point of care during the vaccination session. Moreover, after the Law n. 119/2017, pharmaceutical companies have advertised their vaccine products nationwide more than in the past. In particular, vaccination campaigns for which the necessary authorization was requested to the Ministry of Health were 27 during the years 2014–2016, but increased three times, up to 84, during the years 2017–2019. These campaigns regarded mostly recommended vaccinations available in Italy. As for *N. meningitidis*, there was a low incidence of invasive meningococcal diseases in Italy in 2017–2019, fluctuating around 0.3 cases/100,000 inhabitants, which may have led to a decrease in the attention of the public. Anyway, outbreaks due to *N. meningitidis* serogroup C occurred in Central and North Italy during 2015–2016 and 2019–2020, respectively [17,18]. 

Our evaluation study has the advantage of covering national VC rates; moreover, the VC rate for the last year considered (2019) was well consolidated and not affected by any data communication delay. Finally, we used a direct measure for VC rates calculating the number of vaccines administered each year divided by the number of the targeted population, both numerators and denominators being reported by each R/PA. Limitations may only be due to some geographical variations in VC rates between R/PA. 

Vaccination mandates generated wide public debate in Italy, amplified by traditional and social media, legal actions by some consumer associations, complaints from vaccination centers for not having sufficient resources to efficiently deliver immunization services, together with protests from parents due to the difficulty of booking vaccination appointments. The Ministry of Health replied to many letters from citizens and local health authorities by giving answers to specific requests, while recommending to the vaccination sites to extend the opening hours of vaccination centers. Recent reviews [10,19,20] have assessed national mandatory vaccination policies and consequent health, political and ethical aspects. Globally, 105 countries (of which 23 are in Europe) out of 193 have a nationwide mandatory vaccination policy and 62 countries impose parental, educational and/or financial penalties against not complying individuals, with several level of severity. Italy is the only country to list temporary loss of child custody among these penalties. Moreover, Italian noncompliance parents can be fined up to EUR 500, with no exemptions for religious beliefs against immunizations. The new PNPV 2020–2022 defines the national goals of the vaccination policy and indicates the actions needed for their pursuit at a local, regional and national level. The implementation of the PNPV 2020–2022 undergoes constant monitoring from the R/AP through the evaluation of a system of indicators, measuring not only the VC rates, but also the achievement of the consequent health prevention objectives. Besides, the new digital national vaccine registry (ANV) was established by Decree of the Ministry of Health on 17 September 2018 and will be operational by summer 2021. ANV will be useful both to monitoring the implementation of the vaccination programs in place in Italy, and to providing information to the European and international bodies in the performance and tasks related to health protection, also through the development of indicators for comparative purposes. According to the results of the ASSET project [19], considering just three relevant childhood vaccinations, mandatory vaccination was not associated with childhood immunization rates in the European Union/European Economic Area (EU/EEA) countries. However, the study did not control for possible determinants of VC and it was likely to be affected by ecological fallacia. Parental reasons to vaccinate children are multifactorial, can change over time and with respect of vaccine type. Finally, we must now consider the COVID-19 pandemic. During 2020, the immunization services were temporarily closed in Italy, public alarm was raised, and some parents decided to postpone scheduled vaccinations because of the social distancing measures in place or fear of contagion. In this context, the legal mandate associated with the permission for school attendance will help to restore and consolidate VC rates in children in the upcoming months before the reopening of schools and the next fall.

## 5. Conclusions

In Italy, the 95% value indicated by the World Health Organization (WHO) as the minimum vaccination coverage threshold to guarantee herd immunity has almost been reached after a 3-year period of compulsory broad vaccination policy in children. Our data highlight how the mandatory act may have contributed to a rapid and significant rise in VC rates in childhood. In 2019, VC levels of 95% were reached for nine of the compulsory vaccines, but not for chickenpox, which can also be administered separately from MMR. The PNPV 2017–2019, followed by law reinforcement and the new PNPV 2020–2022, has certainly been an important point in the progress of vaccination policies in Italy for having introduced the concepts of fairness of supply in all R/AP.

Although the Law n. 119/2017 have provided protection for the most, it has caused concern and opposition among others. Our 3-year evaluation allows one to conclude with a positive assessment with regard to the level of VC rates was achieved. However, possible limits of this approach and alternative or complementary options should not be completely ruled out. Besides mandates, education programs promoting responsible behavior and improvement of vaccine literacy should also be considered. Moreover, it should be taken into account that limiting incompletely and unvaccinated children from accessing childcare settings, while reducing the spread of infectious diseases in these sites, might favor potential clustering in informal childcare arrangements, providing new reservoirs for outbreaks. Finally, imposing penalties on vaccine rejecters may not be a solution, and there is some evidence that larger gains may be obtained with less restrictive options [21,22]. The payment of the bill also removes the incentive for parents to discuss vaccine issues with a health professional, or can introduce considerable conflict in the doctor–patient relationship in case of parents seeking vaccination exemptions.

While the legal mandate will continue to support the consolidation of VC rates, especially after the COVID-19 pandemic crisis, vaccine-related education programs, together with relevant information and communication strategies, should be improved to strengthen vaccination behavior and inclusiveness.

## Figures and Tables

**Table 1:** Vaccination coverage (VC) rates (%) registered for mandatory vaccinations, plus chickenpox at 24 months and 6 years of age, stratified by vaccine type and year of administration, along with the percentage differences between 2019 and 2016 rate. Italy, 2014 to 2019.

Target Age Group	VPD	VC Rates (%) by Year of Vaccine Administration						% Difference (2019 vs. 2016)	Denominators 2016–2019	*p*-Value *
		2014	2015	2016	2017	2018	2019			
24 months	Polio	94.71	93.43	93.33	94.6	95.09	95.01	+1.68	461,158–431,723	<0.001
	Measles	86.74	85.29	87.26	91.84	93.22	94.49	+7.23	431,187–429,338	<0.001
	Chickenpox	36.64	30.73	46.06	45.62	74.23	90.50	+44.44	187,107–411,206	<0.001
6 years	Polio	89.18	87.59	85.74	88.69	90.71	88.62	+2.88	482,442–473,491	<0.001
	Measles	87.72	82.98	82.24	85.74	89.2	87.58	+5.34	462,739–467,940	<0.001

* Chi-square test (2019 vs. 2016); VPD: Vaccine-preventable disease; VC: Vaccination coverage; Polio and measles are considered as proxy for the hexavalent and trivalent vaccinations, respectively; Data source: the Italian Regions/Autonomous Provinces provided data.

**Table 2:** Vaccination coverage (VC) rates (%) registered for the recommended vaccinations at 24 months of age, stratified by vaccine and year of administration, along with the percentage differences between 2019 and 2016 rate. Italy 2014 to 2019.

Target Age Group	VPD	VC Rates (%) by Year of Vaccine Administration						% Difference (2019 vs. 2016)	Denominators 2016–2019	*p*-Value *
		2014	2015	2016	2017	2018	2019			
24 months	Men B	n.a.	n.a.	14.72	38.57	46.06	68.98	+54.26	60,011–313721	<0.001
	Men C	73.94	76.62	80.67	82.64	84.93	79.44	−1.23	398,590–361,288	<0.001
	Men ACWY	n.a.	n.a.	18.28	28.56	45.82	46.58	+28.3	74,527–211,836	<0.001
	PNC	87.46	88.73	88.35	90.9	91.89	92.00	+3.65	436,579–418,380	<0.001
	Rotavirus	n.a.	n.a.	10.55	14.36	19.44	26.15	+15.6	40,827–118,930	<0.001

* Chi-square test (2019 vs. 2016); VPD: Vaccine-preventable disease; VC: Vaccination coverage; Men B, C and ACWY: *N. meningitidis* serogroup B, C and ACW135Y; PNC: *S. pneumoniae*; n.a.: data not available; Data source: the Italian Regions/Autonomous Provinces provided data.

## Data Availability

Data supporting reported results can be found at http://www.salute.gov.it/portale/documentazione/p6_2_8_3_1.jsp?lingua=italiano&id=20 (accessed on 5 June 2021).
